# Diverticulectomy in the Management of Intradiverticular Bladder Tumors: A Twelve-Year Experience at a Single Institution

**DOI:** 10.1155/2016/2345306

**Published:** 2016-03-15

**Authors:** Ali Bourgi, Elias Ayoub, Sleiman Merhej

**Affiliations:** Department of Urology, Hotel-Dieu de France University Hospital, Boulevard Alfred Naccache, Achrafieh, P.O. Box 166830, Beirut, Lebanon

## Abstract

*Purpose*. In this retrospective case review we analyze the outcomes of patients treated for intradiverticular bladder tumors (IDT).* Materials and Methods*. A retrospective case review was done between January 2002 and May 2014 in Hotel-Dieu de France hospital. The series included 17 patients diagnosed with IDT, all males with a mean age of 49.8 years.* Results*. One patient was treated with tumor resection and adjuvant BCG instillation with no recurrence on follow-up cystoscopies and urine cytologies. 64% of patients were treated by diverticulectomy. Mean follow-up time was 38.7 months. At the end of the follow-up, 81% were disease-free. One patient had a radical cystectomy 6 months after diverticulectomy for recurrent high grade tumor; another one had a nodal metastasis 10 months after diverticulectomy and was managed with chemotherapy. 29% of patients were treated with radical cystectomy. Mean follow-up time was 28.4 months. No recurrence was documented on annual CT scans.* Conclusions*. Our data support a conservative approach for tumors confined to the bladder diverticulum, even in high grade or in the presence of CIS provided complete removal is feasible and close follow-up is ensured.

## 1. Introduction

Bladder diverticula are outpouchings of bladder wall caused by either congenital or acquired defects.

They are usually thin-walled with a narrow neck and lack the muscularis propria layer. A subset of these lesions, however, may be complicated with inflammation, calculus, infection, and malignancy [1].

Intradiverticular bladder carcinoma is a rare entity with only few studies reported in the literature making its diagnosis and management a unique challenge. It was first described by Targett in 1896 [2] and accounts for approximately 1.5% of bladder tumors. The most common intradiverticular malignancy is urothelial carcinoma [3].

Diagnosis is usually made using a similar fashion to any bladder tumor. Tests include urine cytology, imaging, cystoscopy, and histopathological examination of a resected specimen [4].

Due to its rarity, intradiverticular carcinoma remains infrequently encountered in general practice. Indeed, etiological hypothesis about relationship between bladder diverticula and tumors, histopathologic features, and clinical outcomes of intradiverticular bladder carcinoma are not well investigated, with only six studies of notable size (more than 10 patients) in the current literature, each including less than 40 patients [3].

Treatment modalities range from tumor resection followed by adjuvant intravesical chemotherapy or BCG immunotherapy to diverticulectomy or partial cystectomy and finally radical cystectomy for high grade tumors. Recommendations for intradiverticular bladder tumor management were published by CCFAU in 2012 (Cancer Committee of the French Association of Urology) [3].

In this study, we aimed to review a twelve-year experience in the management of 17 patients with intradiverticular bladder carcinoma between 2002 and May 2014 by characterizing the histopathologic features and comparing clinical outcomes using different treatment modalities including diverticulectomy and radical cystectomy.

## 2. Material/Methods

A retrospective cases review was done between January 2002 and May 2014 in our center (Hotel-Dieu de France university hospital).

During this period, seventeen patients, diagnosed with intradiverticular bladder carcinoma, were selected.

Medical records were reviewed including demographic factors, symptomatology, histological features, and hospital stay.

Follow-up was reviewed with concerning physician. Urine cytology and cystoscopy results were collected and reviewed. Patients were contacted by phone and reevaluated in order to complete missing information.

## 3. Results

All selected patients were of male gender. The mean age was 49.8 years (55–86).

Painless hematuria was the main presenting symptom in 70% of these patients.

Only one patient was treated with tumor resection and adjuvant BCG instillation with no recurrence on follow-up cystoscopies and urine cytologies.

The remaining patients were distributed into two groups depending on the treatment received.

The first group consisted of 11 patients who were treated by open diverticulectomy. In this group, five patients had a high grade urothelial tumor, 3 patients had a CIS, and 3 had a low grade urothelial tumor.

Mean hospital stay was 7.18 days; no complications were documented postoperatively; mean follow-up time was 38 months.

Nine patients (81.81%) were disease-free at the end of follow-up with a mean disease-free survival of 33.63 months. In addition, one patient had a radical cystectomy 6 months after diverticulectomy for recurrent high grade tumor; another one had a ganglionic metastasis 10 months after diverticulectomy and had undergone chemotherapy. Five of seven (71%) patients with invasive tumors and treated with diverticulectomy alone were disease-free at the end of the follow-up.

The other group consisted of five patients who were treated by radical cystectomy. One patient had an adenocarcinoma, 3 had low grade urothelial tumors, and one had a high grade urothelial tumor. Mean hospital stay in this group was 13.2 days; postoperative complications included urinary infections in 2 patients and severe metabolic acidosis in one patient; mean follow-up time was 28 months. No recurrence was documented on annual CT scans.

Clinicopathological characteristics of patients and data relating to intradiverticular bladder tumor staging and survival are summarized in Tables 1 and 2.

## 4. Discussion

Intradiverticular bladder carcinomas are malignant epithelial neoplasms arising within a diverticulum of urinary bladder. Their incidence ranges between 0.8 and 10% [3]. These tumors usually occur in the aged patients with bladder outlet obstruction, rarely on congenital diverticula. Most patients present with macroscopic or microscopic hematuria (up to 90%); others may experience irritative or obstructive symptoms or may be asymptomatic [5].

There is a well-documented relationship between urinary bladder diverticula and bladder cancer.

It is thought that stasis of urine in the bladder diverticulum produces chronic mucosal irritation and prolonged exposure to urinary carcinogens, thus increasing the risk of malignancies of the diverticulum epithelial lining [6, 7].

Absence of muscularis propria layer may in theory predispose intradiverticular tumors to a greater risk for bladder wall infiltration and possibly spreading into adjacent organs; however the aggressiveness of these tumors, likeliness of spreading, and their clinical outcomes remain poorly characterized [8].

Imaging diagnosis and staging rely on CT scan; however this is limited by its inability to resolve the different layers of the bladder wall. Magnetic resonance (MR) imaging of the bladder provides a better gross assessment of tumor depth, especially when performed with Gadolinium injection. Postcontrast enhancement allows further evaluation of tumor extent and spread to adjacent organs [9].

Lacking a muscle layer, diverticular walls tumors present a unique difficulty in staging. Indeed, some authors suggest skipping the T2 stage altogether when staging diverticular tumors [10].

In their study, Zhong et al. reviewed 22 patients with intradiverticular bladder carcinoma. Results showed no statistical difference in disease-free survival or overall survival between noninvasive and invasive tumors within approximately 3 years of follow-up. They also confirmed previous observation that intradiverticular carcinomas are often associated with a hypertrophic layer of muscularis mucosae that can potentially confound tumor staging and that the absence of a muscularis propria layer may not necessarily predispose T1 tumors to more aggressive disease [8].

In their study, Baniel and Vishna reviewed the clinical outcome of 8 patients with primary intradiverticular transitional cell carcinoma who were treated conservatively (TURB, diverticulectomy, or partial cystectomy followed with either Bacille Calmette-Guerin (BCG) instillations, Thiotepa instillations, or postoperative irradiation). After a mean follow-up duration of 6 years, six of eight patients were disease-free. Similar results were observed in our study where 81.81% of patients treated conservatively were disease-free after a median follow-up of 38 months [11].

**Figure 1:**
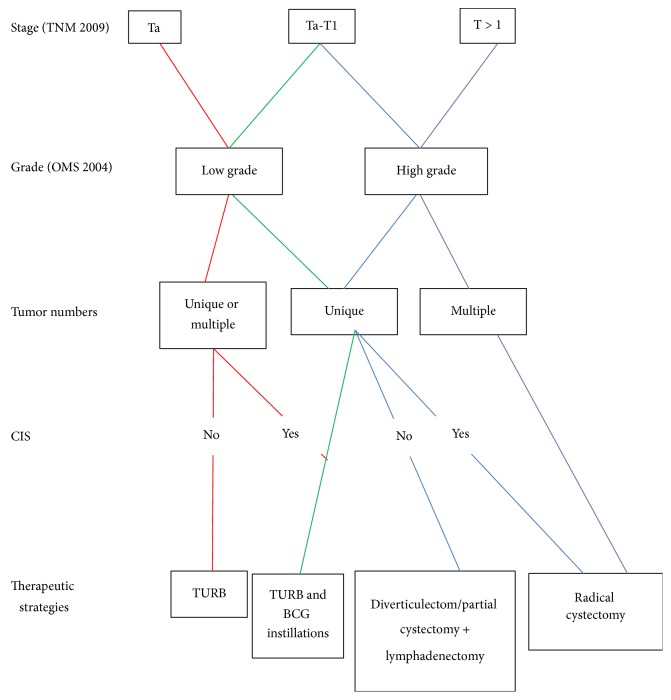
Management of intradiverticular bladder tumor: review of the cancer committee of the French Association of Urology.

Until this day, no clear guidelines were published concerning the management of intradiverticular bladder tumor. The only treatment strategy was proposed and published in 2012 by the review of the cancer committee of the French Association of Urology (Figure 1). Golijanin et al. reported an overall survival of 66% at 4.2 years and 75% at 2.7 years, respectively [1, 10]. In our study, we proved that diverticulectomy is an effective and safe treatment option for high grade IDBT with a disease-free survival rate of 71% at the end of a follow-up.

## 5. Conclusion

Diverticulectomy is a safe and effective procedure in the treatment of IDBT. Our data support a conservative approach for tumors confined to the bladder diverticulum, even in high grade or in the presence of CIS provided complete removal is feasible and close follow-up ensues. Further studies on conservative management strategies, impact on quality of life, and prognosis would be helpful in guiding patient and physician decisions on treatment choice and postoperative surveillance strategies.

## Figures and Tables

**Table 1:** Clinicopathological characteristics of patients.

Variable	Number of patients (%)
*Clinical stage*	
Noninvasive	9 (53)
Invasive	8 (47)
*CIS*	
Present	3 (18)
Absent	14 (82)
*Primary mode of treatment*	
Transurethral resection + BCG	1 (6)
Diverticulectomy	11 (65)
Radical cystectomy	5 (29)

**Table 2:** Data relating to intradiverticular bladder tumour staging, follow-up, and survival.

Treatment received	Number of patients	Local recurrence (%)	Developed metastases (%)	Follow-up period (months)
BCG instillation	1	0	0	100
Open diverticulectomy	11	1 (9%)	1 (9%)	38
Radical cystectomy	5	0	0	28
